# Ovarian Fibroma With Minor Sex Cord Elements and Luteinized Cells in an 81‐Year‐Old Woman: A Case Report With Molecular Findings and Review of the Literature

**DOI:** 10.1155/crip/3850274

**Published:** 2026-11-01

**Authors:** Akbar Ali, Shivam Gandhi, Christopher Potts, Nathanial Branscome, Sheharyar Raashid, Mariam Ghafoor

**Affiliations:** ^1^ Marshall University Joan C. Edwards School of Medicine Huntington West Virginia USA marshall.edu

**Keywords:** FOXL2, luteinized cells, minor sex cord elements, next-generation sequencing, ovarian fibroma, sex cord-stromal tumor

## Abstract

**Background:**

Ovarian fibroma with minor sex cord elements (MSCE) is a fibroma or fibrothecoma containing scattered sex cord‐type elements that occupy less than 10% of the tumor area. First described by Young and Scully in 1983, it is not recognized as a separate entity in the current World Health Organization classification, and its behavior follows that of the underlying stromal tumor. About two dozen ovarian cases have been published, a number that probably reflects under‐reporting rather than true rarity. Reported patients range from 13 to 81 years of age.

**Case:**

An 81‐year‐old woman with a remote total hysterectomy with ovarian preservation presented with abdominal discomfort, constipation, and a pelvic mass that computed tomography initially described as uterine. Pelvic ultrasonography showed a 10.6 cm complex cystic mass adjacent to the vaginal cuff. Robotic‐assisted bilateral salpingo‐oophorectomy removed a 9 cm right ovarian mass. Histology showed a fibroma with scattered tubules, trabeculae, and nests of sex cord‐type cells and a few clusters of luteinized cells, together less than 10% of the tumor; the sex cord elements expressed SF‐1, WT1, calretinin, and inhibin. A targeted next‐generation sequencing panel performed by the consulting reference laboratory showed no reportable variants, including no *FOXL2* c.402C>G mutation, and microsatellite stability. Pelvic washings were negative. She had no clinical signs of hormone excess before surgery; hot flashes reported at follow‐up were not investigated biochemically and have several possible explanations. There was no recurrence at 8 months.

**Conclusion:**

This case adds an octogenarian patient and targeted sequencing data to the limited published experience with ovarian fibroma with MSCE. Recognition of the minor sex cord component did not change management; its practical importance lies in excluding adult granulosa cell tumor, for which adequate sampling and, when needed, *FOXL2* mutation analysis are useful.

## 1. Introduction

Ovarian fibroma with minor sex cord elements (MSCE) was described by Young and Scully in 1983 as a predominantly fibromatous or thecomatous tumor containing small nests, tubules, or trabeculae of sex cord‐type cells occupying less than 10% of the tumor area [1]. Ovarian fibromas are common, and a minor sex cord component is easily overlooked or left unreported because it carries no prognostic significance. The small number of published cases—about two dozen ovarian examples in our review (Table 1)—therefore probably reflects under‐recognition and under‐reporting rather than a truly rare tumor.

**Table 1:** Reported cases of ovarian fibroma with minor sex cord elements.

Reference	Year	Age (yrs)	Presentation	Tumor side/size	Hormonal activity	Associated endometrial pathology	Treatment	Outcome
Young & Scully [1]	1983	16–65	Variable; incidental to mass effect	Variable; 1–10 cm	Variable; some active	Endometrial adenocarcinoma in 2 of 7 cases	Surgical excision	Benign; no recurrence
Arai et al. [2]	1999	57	Postmenopausal bleeding, abdominal mass	Right ovary; 6.0 × 5.0 cm	Testosterone 231 ng/dL and estradiol 45 pg/mL elevated without androgenic signs; testosterone normal 3 days after surgery	Simple hyperplasia	TAH‐BSO, omentectomy	No recurrence at 2 years 11 months
Yang et al. [3]	2001	58	Ovarian mass; coexisting mucinous cystadenoma	5 cm ovary with 1.5 cm stromal nodule	Not stated	Not stated	Surgical excision	Not stated
Lee & Ahmed [4]	2003	69	Pelvic mass, postmenopausal bleeding	Right ovary; 13 × 8.5 × 6 cm	Hyperestrogenic	Complex atypical hyperplasia with focal well‐differentiated endometrioid adenocarcinoma	TAH‐BSO	Fibrosarcomatous transformation—sole reported case of malignant degeneration
Mandal et al. [5]	2007	45	Dysfunctional uterine bleeding/menorrhagia; prolapse (incidental finding)	Right ovary; normal sized ovary; tumor 1.8 cm	Not active	None	TAH‐BSO (for prolapse)	Benign; no recurrence
Kawatra et al. [6]	2008	65	Postmenopausal bleeding, abdominal pain	Right ovary; 5 × 4 × 2 cm	Hyperestrogenic (clinical)	Simple glandular hyperplasia without atypia + endometrial polyp	TAH‐BSO	Benign; no recurrence
Sherwani et al. [7]	2011	52	Right lower abdominal mass/pain for 4 years; incidental finding	Right ovary; Size not reported	Not specified; possibly silent	None reported	BSO	Benign; no recurrence
Kumar S et al. [8]	2013	79	Postmenopausal bleeding and lower abdominal pain	Left ovary; 20 × 10 cm	Hyperestrogenic	Well‐differentiated endometrioid adenocarcinoma	TAH‐BSO, infra‐colic omentectomy, pelvic lymphadenectomy	Benign ovarian tumor; carcinoma managed separately
Sood et al. [9]	2014	13	Oligomenorrhea, virilization, abdominal mass	Left ovary; 10 cm	Androgenic (virilization; elevated serum testosterone, normalized after surgery)	Not stated	Unilateral salpingo‐oophorectomy	Virilization regressed at 4 months; then lost to follow‐up
Shilpa et al. [10]	2016	55	Lower abdominal heaviness, postmenopausal bleeding	Right ovary; 9 × 7.5 × 5 cm (gross)/10 × 11 cm	Hyperestrogenic	Well‐differentiated endometrioid adenocarcinoma (Grade 1) + complex atypical hyperplasia	TAH‐BSO	Benign ovarian tumor; carcinoma managed separately
Mathur et al. [11]	2018	52	Irregular vaginal bleeding for 1.5 years	Right ovary; 5.0 × 3.5 × 2.5 cm	Estrogenic (clinical); luteinized thecal cells	Simple hyperplasia without atypia	Hysterectomy, right salpingo‐oophorectomy	Asymptomatic at 4 months
Mahendran & Kanchana [12]	2020	70	Postmenopausal bleeding with abdominal mass	Left ovary; 7 × 6 × 4 cm	Estrogenic (proliferative endometrium with polyp in a 70‐year‐old postmenopausal woman)	Proliferative endometrium with benign endometrial polyp	TAH‐BSO	Benign
Kumar R et al. [13]	2023	60	Postmenopausal bleeding	Left ovary; 5.5 × 4.5 × 2.5 cm (cellular fibroma)	Estrogenic (clinical)	Moderately differentiated endometrioid adenocarcinoma	TAH‐BSO, pelvic lymph node sampling	Lost to follow‐up
Miki et al. [14]	2024	81	Constipation, pelvic mass; prior hysterectomy and left salpingo‐oophorectomy	Right ovary; 10.4 × 9.6 cm	Not described	Not applicable (prior hysterectomy)	Laparotomy, right salpingo‐oophorectomy	No recurrence at 2 years
Rathore et al. [15]	2026	56	Intermittent postmenopausal bleeding, lower abdominal discomfort	Left ovary; 3.5 cm	Estrogenic (clinical); serum inhibin normal	Endometrial polyp	TAH‐BSO	Well at 2 months
Present case	2025	81	Pelvic mass, abdominal discomfort, constipation	Right ovary; 9 cm	No clinical signs preoperatively; hot flashes at 8 months, not investigated; no hormone levels	Not applicable (prior hysterectomy)	Robotic BSO	Benign; no recurrence at 8 months

Some reported tumors have been associated with estrogen excess, including endometrial hyperplasia and endometrioid adenocarcinoma [4, 6, 8, 10, 11, 13], and two androgen‐producing examples with elevated serum testosterone have been documented [2, 9]; others were incidental and hormonally inert [5, 7]. Behavior follows that of the underlying stromal tumor, and the only reported malignant change is a single fibrosarcoma arising in a fibrothecoma with MSCE [4].

The main practical relevance of the minor sex cord component is diagnostic: a fibroma with conspicuous sex cord elements can be mistaken for adult granulosa cell tumor, a low‐grade malignancy that warrants long‐term surveillance. Immunohistochemistry and, in difficult cases, *FOXL2* mutation analysis help make this distinction [16–18]. Molecular data specific to fibroma with MSCE are limited to *FOXL2* mutation testing and loss‐of‐heterozygosity studies in a few cases [18, 19].

We report ovarian fibroma with MSCE and luteinized cells in an 81‐year‐old woman, add targeted next‐generation sequencing (NGS) results to the limited molecular data, and summarize the published ovarian cases identified through a structured literature search.

## 2. Case Presentation

### 2.1. Clinical History and Presentation

The patient was an 81‐year‐old woman, gravida 4 para 4, with a complex medical history including paroxysmal atrial fibrillation (on rivaroxaban), chronic kidney disease Stage 3a (baseline creatinine 1.1–1.3 mg/dL), hypertension, chronic obstructive pulmonary disease, obstructive sleep apnea (on CPAP with suboptimal compliance), history of cerebrovascular accident without residual deficits, and a remote total hysterectomy with ovarian preservation. Her CHA₂DS₂‐VASc score was 7, reflecting high cardiovascular risk.

She presented to the emergency department with lower abdominal discomfort, constipation, and an episode of gross hematuria. Noncontrast computed tomography (CT) at an outside facility described an “enlarged, heterogeneous, and lobulated uterus”; because the patient reported her prior hysterectomy, the mass was presumed to be adnexal and she was referred to gynecology.

The patient was referred for transvaginal pelvic ultrasonography, which identified a complex cystic area measuring 10.6 × 6.0 × 7.4 cm in close proximity to the vaginal cuff, with heterogeneous internal echogenicity and no significant vascularity on color Doppler imaging (Figure 1). Pelvic examination by gynecologic oncology revealed a large, firm, mobile mass palpable in the lower abdomen. Given the size, complexity, and patient′s age, surgical excision was recommended for definitive diagnosis and management. The patient was counseled regarding the possibility of malignancy, potential staging procedures, and possible conversion to laparotomy.

**Figure 1:**
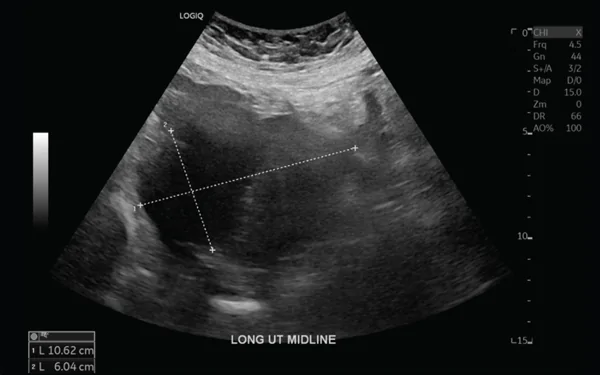
Representative pelvic ultrasonography images demonstrating the complex cystic mass adjacent to the vaginal cuff.

### 2.2. Surgical Intervention

The patient underwent robotic‐assisted bilateral salpingo‐oophorectomy. Intraoperative findings included a large, complex right ovarian mass measuring approximately 8–9 cm with adhesions to the posterior cul‐de‐sac. The uterus and cervix were confirmed to be surgically absent. The left fallopian tube and ovary appeared grossly normal. No other intraperitoneal masses, lesions, or peritoneal implants were identified. Pelvic washings were obtained for cytology. After extensive adhesiolysis, the right adnexa and mass were placed intact in an Endo Catch bag; within the bag, fluid was drained from the mass, which was then removed through an enlarged port site and fragmented during extraction. The left tube and ovary were removed separately. Estimated blood loss was 10 mL, and there were no intraoperative complications.

### 2.3. Pathologic Findings

#### 2.3.1. Gross Examination

The specimen was received fragmented. Serial sectioning showed tan‐pink, firm, glistening cut surfaces with poorly defined cystic spaces. No necrosis was identified.

#### 2.3.2. Microscopic Findings

The H&E slides as well the blocks were sent to the reference laboratory for consultation. Sections of the right ovarian mass demonstrated a predominantly spindle cell proliferation arranged in a storiform and loosely fascicular architecture (Figure 2). The neoplastic cells showed moderate amounts of pale eosinophilic cytoplasm and bland nuclei within an edematous to fibrocollagenous background, supportive of fibroma. Interspersed throughout the fibromatous stroma were scattered tubules, trabeculae, and small nests of cells with scant pale basophilic to clear cytoplasm and elongated nuclei, consistent with sex cord elements. Additionally, a few clusters of luteinized cells with abundant eosinophilic cytoplasm were present. The cystic spaces were lined by cuboidal cells. The sex cord and luteinized components together represented less than 10% of the well‐sampled tumor. No significant cytologic atypia, mitotic activity, or necrosis was identified. The tumor had a mostly normocellular to hypocellular appearance, typical of fibroma.

**Figure 2:**
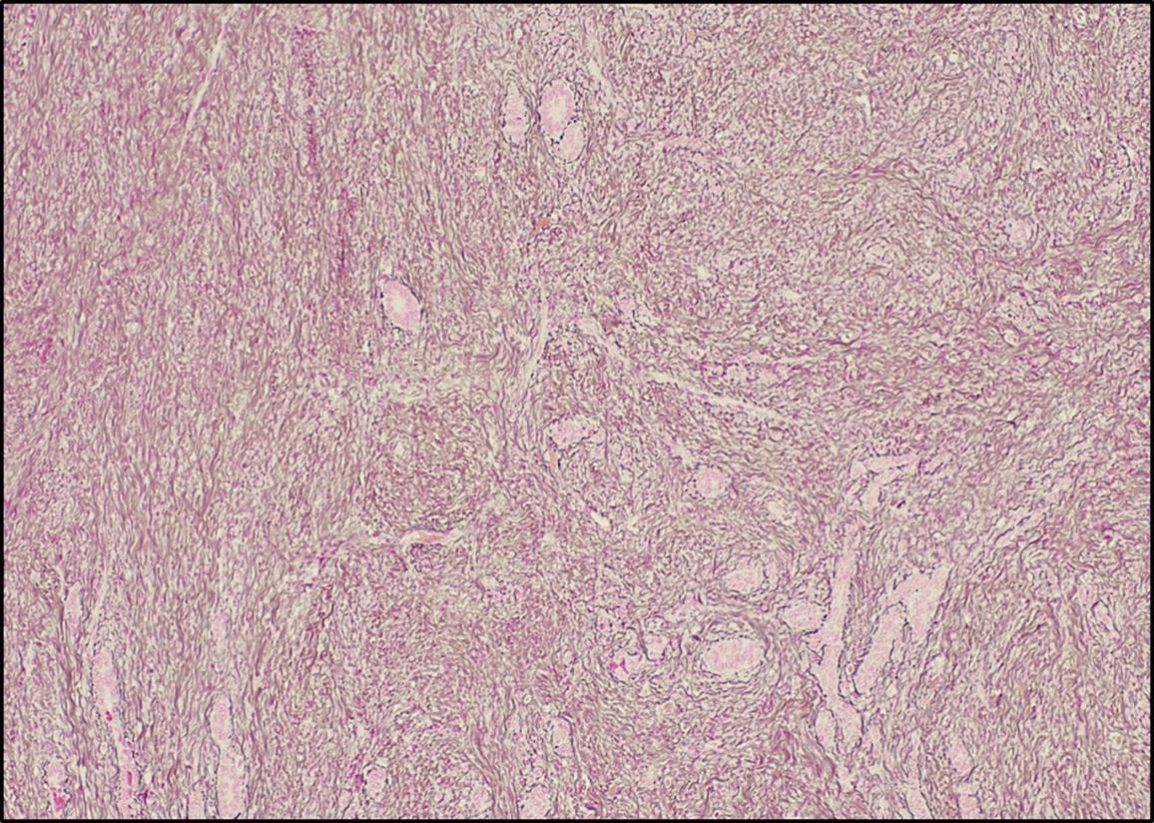
Hematoxylin and eosin (H&E) stained sections demonstrating the fibromatous stroma with storiform and fascicular growth patterns with luteinized cells, at low power (2a) and high power (2b); interspersed sex cord cells identified as a minor component, at low power (2c) and high power (2d).

#### 2.3.3. Immunohistochemistry

A comprehensive immunohistochemical panel was performed (Figure 3). Reticulin highlighted the group of sex cord elements (showed individual pericellular staining pattern in the fibromatous component). The nests of sex cord cells showed diffuse expression of SF‐1 (steroidogenic factor 1), WT1 (Wilms tumor 1), focal expression of calretinin and patchy expression of inhibin, supporting sex cord‐stromal differentiation. Epithelial membrane antigen (EMA) was negative in these sex cord elements, excluding epithelial differentiation. The overall immunoprofile was consistent with a sex cord‐stromal tumor, specifically ovarian fibroma with MSCE.

**Figure 3:**
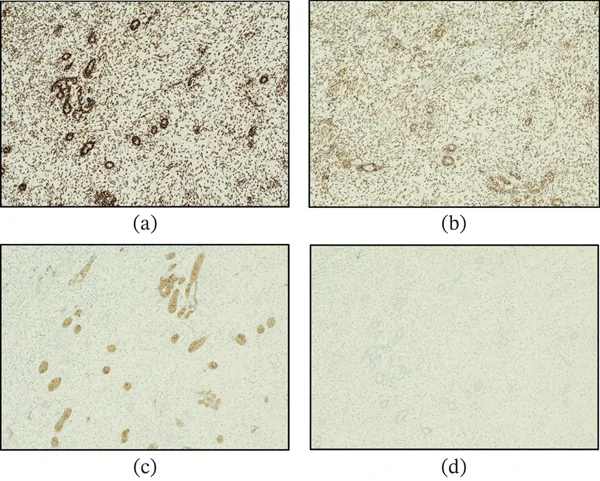
Representative immunohistochemical stains demonstrating (a) diffuse SF‐1 (steroidogenic factor 1) expression within both the fibromatous and sex cord components, confirming sex cord‐stromal differentiation; (b) diffuse WT1 (Wilms tumor 1) expression throughout the tumor; (c) patchy inhibin expression selectively highlighting the sex cord elements against a negative fibromatous background; and (d) focal calretinin expression highlighting the sex cord elements.

#### 2.3.4. Molecular Testing

NGS was performed, which interrogated coding regions and exon/intron boundaries of genes relevant to gynecologic neoplasms, including sex cord‐stromal tumor–relevant genes (*FOXL2*, *DICER1*, and *STK11*) and common cancer genes (*AKT1*, *APC*, *ARID1A*, *ARID1B*, *ATM*, *ATR*, *ATRX*, *BAP1*, *BARD1*, *BRAF*, *BRCA1*, *BRCA2*, *BRIP1*, *CDK12*, *CDKN2A*, *KRAS*, *PIK3CA*, *PTEN*, *TP53*, and others). It showed no reportable sequence variants. Microsatellite status was also assessed to be stable (MSS). The absence of somatic mutations, including the absence of *FOXL2* mutations (characteristic of adult granulosa cell tumors), supported the diagnosis of ovarian fibroma with MSCE rather than granulosa cell tumor or other sex cord‐stromal neoplasm.

#### 2.3.5. Cytology

Pelvic washing cytology revealed benign mesothelial cells and lymphocytes with no evidence of malignancy.

#### 2.3.6. Final Pathologic Diagnosis

Right ovary and fallopian tube: ovarian fibroma with MSCE and luteinized cells. Left ovary and fallopian tube: benign; no pathologic abnormality identified.

### 2.4. Postoperative Course

The patient tolerated the procedure well and was discharged on postoperative Day 1. At her first postoperative visit (approximately 6 weeks postsurgery), she reported resolution of the pelvic pressure and abdominal discomfort that had prompted initial evaluation.

The patient had no postmenopausal bleeding, virilization, or hirsutism before surgery. At the 8‐month follow‐up visit she reported hot flashes; these were not evaluated further and no hormone levels were obtained before or after surgery.

During the follow‐up period she was hospitalized for paroxysmal atrial fibrillation, which was treated with catheter ablation and changes to her antiarrhythmic and rate‐control medications. At the most recent follow‐up (8 months after surgery), there was no clinical or imaging evidence of recurrence, and routine surveillance was planned.

## 3. Discussion

### 3.1. Classification and Published Cases

Fibroma with MSCE is defined by scattered sex cord elements—tubules, trabeculae, or nests of epithelioid cells—within a predominantly fibromatous or thecomatous stroma and occupying less than 10% of the tumor area [1, 5]; more extensive sex cord differentiation indicates another tumor, such as adult granulosa cell tumor or sex cord tumor with annular tubules. In the current World Health Organization classification, stromal tumor with MSCE is not a separate entity: these tumors are classified with the underlying fibroma or thecoma, and the MSCE are not considered to alter prognosis [20]. Because ovarian fibromas are common and the minor component is clinically inconsequential, it is probably often unreported, and the published experience likely underestimates its true frequency.

To summarize the published experience, we searched PubMed/MEDLINE from inception to October 3, 2026, without date restrictions, using two queries: “minor sex cord elements”[tiab], and (“minor sex cord”[tiab] OR “sex cord elements”[tiab]) AND (fibroma[tiab] OR thecoma[tiab] OR fibrothecoma[tiab] OR “stromal tumor”[tiab] OR “stromal tumour”[tiab]). The searches returned 38 unique records. We retained English‐language reports of ovarian fibroma, fibrothecoma, or stromal tumor with MSCE that described individual patients, and excluded testicular, uterine, and extraovarian tumors, ovarian fibromatosis, tumors ultimately classified as another entity, and non‐English reports. Two reports in journals not indexed in MEDLINE, identified during manuscript preparation, were also included [7, 12].

The included cases are summarized in Table 1. Patients have ranged from 13 to 81 years of age [1, 9, 14]. Our patient and the 81‐year‐old woman reported by Miki et al. [14] are the oldest described; that case was strikingly similar—a woman with a remote hysterectomy who presented with constipation and a 10 cm pelvic mass, with sex cord elements comprising less than 1% of the tumor and no recurrence at 2 years. Advanced age has no known bearing on the behavior of this tumor.

### 3.2. Imaging and Gross Correlation

The initial CT report attributed the mass to the uterus; because the patient reported her hysterectomy, this was corrected promptly and did not affect management.

Ultrasonography showed a complex cystic mass, whereas grossly the tumor was firm with only poorly defined cystic spaces. Large fibromas commonly undergo stromal edema and cystic degeneration, which produce cystic components on imaging, and the stroma of this tumor was edematous in areas. In addition, the mass was drained within the retrieval bag before extraction, so fluid‐filled spaces visible on imaging would have been collapsed by the time of gross examination. Microscopically, the residual cystic spaces were lined by cuboidal cells.

### 3.3. Pathologic Features and Differential Diagnosis

The dominant component of fibroma with MSCE is a bland spindle cell proliferation resembling conventional ovarian fibroma, within a collagenous or edematous stroma. The MSCE appear as tubules, trabeculae, small nests, or cords of epithelioid to cuboidal cells with pale cytoplasm and round to oval nuclei. Although the defining criterion is that they occupy less than 10% of the tumor area, some authors have further specified that individual aggregates should not exceed approximately 0.45 mm [10]. Recognition of the minor sex cord component did not change management in this patient: bilateral salpingo‐oophorectomy was curative, no staging, adjuvant therapy, or tumor‐marker surveillance was indicated, and follow‐up was the same as for any benign ovarian fibroma. The practical value of adequate sampling lies in classification. Sampling confirms that the sex cord component is truly minor and cytologically bland and thereby excludes adult granulosa cell tumor, a diagnosis that would carry a risk of late recurrence and warrant prolonged surveillance; conversely, limited sampling of a tumor with a more extensive granulosa cell component could lead to underdiagnosis. Kumar et al. advised long‐term follow‐up for patients with this tumor because of this uncertainty [8]; when the sex cord component is clearly minor and *FOXL2* mutation testing is negative, as here, routine follow‐up appears sufficient.

In our case, the sex cord elements were identified on H&E and further characterized by immunohistochemistry, with diffuse WT1 and SF‐1 expression and variable calretinin and inhibin expression, markers commonly expressed in sex cord‐stromal tumors. Reticulin staining highlighted the nests of sex cord cells. Clusters of luteinized cells with abundant eosinophilic cytoplasm were also present; luteinized cells have been described in other fibromas with MSCE and are a potential source of steroid hormone production [7, 10, 11].

The principal differential is adult granulosa cell tumor. Granulosa cell tumors are composed predominantly of sex cord cells, far exceeding the 10% threshold, and show microfollicular, trabecular, or insular architecture with nuclear grooves and Call–Exner bodies, diffuse inhibin and calretinin expression, and in about 95% of cases, the *FOXL2* c.402C>G (p.C134W) mutation [17], which was absent here and has not been found in cellular fibromas, including three with MSCE [18]. The pericellular reticulin pattern of the dominant fibromatous component, with group‐investing reticulin confined to the minor sex cord nests, further supports fibroma with MSCE. Other considerations include Sertoli–Leydig cell tumor (younger patients, extensive Sertoli tubules with Leydig cells, frequent virilization), sclerosing stromal tumor (pseudolobular architecture with staghorn vessels), thecoma (sheets of luteinized stromal cells without discrete sex cord formations), and, particularly in older patients, metastatic carcinoma (excluded by negative EMA and positive sex cord markers).

### 3.4. Hormonal Activity

Some fibromas with MSCE have been associated with estrogen excess—endometrial hyperplasia, endometrioid adenocarcinoma, or postmenopausal bleeding—attributed to thecomatous or luteinized stromal cells or to the sex cord elements themselves [4, 6, 8, 10, 11, 13]. Sherwani et al. and Mathur et al. described fibromas with MSCE containing luteinized cells [7, 11], and Sood et al. documented virilization with elevated serum testosterone that normalized after surgery [9]. Arai et al. found elevated serum testosterone and estradiol in a patient with no androgenic signs, with testosterone normalizing within 3 days of surgery [2], showing that hormone production by these tumors can be clinically silent. In most reports, however, hormonal activity was inferred from endometrial findings rather than measured.

Not all of these tumors are hormonally active. Some were incidental findings, and others presented only with a pelvic mass and pressure symptoms, without reported hormonal manifestations [5, 7, 14]. Why some tumors are hormonally active and others are not is unclear; luteinized stromal cells and the sex cord elements themselves, including granulosa‐type cell aggregates, have each been proposed as the source of steroid production [8, 10, 11].

Our patient had no clinical signs of hormone excess before surgery, and endometrial effects could not be assessed because of her prior hysterectomy. She first reported hot flashes at the 8‐month follow‐up visit. The luteinized cells in the tumor are a plausible source of low‐level steroid production, and withdrawal of tumor‐derived estrogen is one possible explanation, but it cannot be established: no hormone levels were obtained before or after surgery, and other explanations are at least as likely. Bilateral salpingo‐oophorectomy also removed the contralateral, histologically normal ovary; the postmenopausal ovary remains hormonally active, contributing to circulating testosterone, a substrate for peripheral estrogen production, even more than 10 years after menopause, and serum testosterone and estrone fall after oophorectomy [21]. The interval of several months between surgery and symptom onset is also atypical for an acute withdrawal effect. Nongynecologic causes are plausible as well: during follow‐up she had paroxysmal atrial fibrillation requiring hospitalization and changes to her cardiovascular medications, she takes hydralazine, a vasodilator that can cause flushing, and she has documented anxiety. We therefore do not attribute her symptoms to the tumor, and we note the observation only as a reason to obtain baseline estradiol and testosterone levels when a luteinized stromal tumor is suspected before surgery.

### 3.5. Molecular Characterization

The diagnosis rests on histology and immunohistochemistry, which are usually sufficient to distinguish fibroma with MSCE from adult granulosa cell tumor. In this case, the slides and blocks were sent to a reference laboratory for expert consultation because of the sex cord component, and the consulting pathologists performed a targeted NGS panel as part of that workup to exclude adult granulosa cell tumor. The panel covered the coding regions and exon/intron boundaries of more than 50 genes relevant to gynecologic neoplasms, including *FOXL2*, *DICER1*, and *STK11*. No reportable variants were found, including the *FOXL2* c.402C>G mutation, and the tumor was microsatellite stable. This agrees with McCluggage et al., who found no *FOXL2* mutation in 22 cellular fibromas, three of which contained MSCE [18], and with earlier work showing loss of heterozygosity at 19p13.3 without *STK11* mutation in fibrothecomas with sex cord elements [19]. FOXL2 immunohistochemistry does not discriminate between these tumors because fibromas also express the protein [18]; when molecular confirmation is needed, single‐gene *FOXL2* mutation testing would usually suffice, and the broader panel adds limited but, to our knowledge, previously unreported data for this tumor.

### 3.6. Clinical Management and Prognosis

The treatment of ovarian fibroma with MSCE is surgical excision. In premenopausal women, fertility‐sparing surgery may be considered if the tumor is unilateral and there is no evidence of malignancy; in postmenopausal women, as here, bilateral salpingo‐oophorectomy is typical. The robotic‐assisted approach resulted in minimal blood loss (10 mL) and an uneventful recovery despite the patient′s age and comorbidities; the specimen was contained in a retrieval bag throughout extraction, although it fragmented during removal.

The prognosis is excellent, consistent with the WHO view that MSCE do not alter the behavior of the underlying stromal tumor [20]; most reports document no recurrence or metastasis [1, 5–8, 10, 12, 14]. The only exception is the case reported by Lee and Ahmed, in which fibrosarcoma arose within a fibrothecomatous tumor with MSCE [4]. In our case, the tumor showed no cytologic atypia, increased mitotic activity, or necrosis.

One additional consideration is surveillance for endometrial pathology when the tumor is hormonally active and the patient has an intact uterus, as estrogen excess from these tumors has been associated with endometrial hyperplasia or carcinoma [8, 10]. This was not applicable here given the patient′s prior hysterectomy. When endometrial assessment is indicated, endometrial sampling (rather than imaging alone) is the appropriate means of evaluation.

### 3.7. Limitations

This report has limitations. No hormone levels were obtained before or after surgery, so the hormonal activity of the tumor cannot be determined. The CT images were not available for review. The cystic spaces could not be fully characterized grossly because the specimen was drained and fragmented. Follow‐up is limited to 8 months, although recurrence of this tumor has not been reported. Our literature review was limited to PubMed and English‐language reports and may have missed cases.

## 4. Conclusion

We report ovarian fibroma with MSCE and luteinized cells in an 81‐year‐old woman, which, with a similar recently reported case [14], represents the oldest age at which this tumor has been described, and we add targeted sequencing results showing no *FOXL2* mutation and microsatellite stability. Recognition of the minor sex cord component did not change management; its practical importance lies in distinguishing the tumor from adult granulosa cell tumor. Postoperative hot flashes were not investigated and have several possible explanations, so hormonal activity of the tumor remains unproven; baseline hormone levels are worth obtaining when a luteinized stromal tumor is suspected before surgery.

## Author Contributions

Akbar Ali, Shivam Gandhi, Christopher Potts, and Nathanial Branscome: conceptualization, writing—original draft, and writing—review and editing. Sheharyar Raashid and Mariam Ghafoor: supervision and writing—review and editing.

## Funding

No funding was received for this manuscript.

## Disclosure

All authors approved the final manuscript.

## Consent

Written informed consent was obtained from the patient for publication of this case report and accompanying images. A copy of the written consent is available for review by the Editor‐in‐Chief of this journal upon request.

## Conflicts of Interest

The authors declare no conflicts of interest.

## Data Availability

Research data are not shared.
